# DNA Methylation and Type 2 Diabetes: Novel Biomarkers for Risk Assessment?

**DOI:** 10.3390/ijms222111652

**Published:** 2021-10-28

**Authors:** Gregory Alexander Raciti, Antonella Desiderio, Michele Longo, Alessia Leone, Federica Zatterale, Immacolata Prevenzano, Claudia Miele, Raffaele Napoli, Francesco Beguinot

**Affiliations:** 1 Department of Translational Medical Sciences, Federico II University of Naples, 80131 Naples, Italy; antonella.desid@gmail.com (A.D.); mi_longo@libero.it (M.L.); aleleone86@libero.it (A.L.); federicazatterale@libero.it (F.Z.); imma.prevenzano@libero.it (I.P.); c.miele@ieos.cnr.it (C.M.); napoli@unina.it (R.N.); beguino@unina.it (F.B.); 2 URT Genomics of Diabetes, Institute of Experimental Endocrinology and Oncology, National Research Council, 80131 Naples, Italy

**Keywords:** biomarkers, DNA methylation, type 2 diabetes, epigenetics, clinical practice

## Abstract

Diabetes is a severe threat to global health. Almost 500 million people live with diabetes worldwide. Most of them have type 2 diabetes (T2D). T2D patients are at risk of developing severe and life-threatening complications, leading to an increased need for medical care and reduced quality of life. Improved care for people with T2D is essential. Actions aiming at identifying undiagnosed diabetes and at preventing diabetes in those at high risk are needed as well. To this end, biomarker discovery and validation of risk assessment for T2D are critical. Alterations of DNA methylation have recently helped to better understand T2D pathophysiology by explaining differences among endophenotypes of diabetic patients in tissues. Recent evidence further suggests that variations of DNA methylation might contribute to the risk of T2D even more significantly than genetic variability and might represent a valuable tool to predict T2D risk. In this review, we focus on recent information on the contribution of DNA methylation to the risk and the pathogenesis of T2D. We discuss the limitations of these studies and provide evidence supporting the potential for clinical application of DNA methylation marks to predict the risk and progression of T2D.

## 1. Introduction

Diabetes mellitus is one of the fastest-growing health emergencies and a leading cause of death worldwide (4.2 million deaths in 2019) [1]. Approximately 463 million people lived with diabetes in 2019. Most of them (about 90%) have type 2 diabetes (T2D) [1]. Projections for 2030 and 2045 predict that adults with diabetes will reach 578 and 700 million, respectively [1].

T2D is a complex, chronic and progressive disorder mainly characterized by a dysregulation of nutrient metabolism (Appendix A) resulting from a combination of modifiable and non-modifiable risk factors [2]. This disorder is most common in older adults, but due to the rising obesity epidemic, even at younger ages, prevalence is increasing in both children and young adults [1]. Increased adiposity, body mass index (BMI) ≥ 25 kg·m^−2^ or abdominal obesity are the most critical risk factors for T2D [3,4,5]. Population aging, economic development and increased urbanization, along with more sedentary lifestyles, including physical inactivity and prolonged television watching, cigarette smoking and consumption of unhealthy foods (e.g., sugar-sweetened beverages and alcohol), are also important to the rise in T2D prevalence [1,6,7,8,9,10].

T2D onset is often preceded by a history of metabolic syndrome, gestational diabetes, polycystic ovary syndrome and the presence of acanthosis nigricans [2]. In addition, T2D is more frequent in certain ethnic and family groups [11,12].

The physical and psychological burden of people living with diabetes and its complications can be effectively managed [13,14,15]. The education and adoption of a healthy lifestyle may also prevent T2D or delay its onset in at-risk individuals [16,17,18].

Up to 90% of T2D cases are potentially preventable by adopting a healthy diet, maintaining a BMI of ≤25 kg·m^−2^, exercising for at least 30 minutes per day, and avoiding cigarettes and alcohol [19,20]. To reach this goal, identifying subjects at high risk for T2D is critical. Screening for T2D is now recommended in at-risk individuals, including adults ≥45 years of age, those who are overweight, obese, pre-diabetic or in individuals who have first-degree relatives with diabetes [21].

Prediction models for T2D, where clinical and laboratory data and information of known risk factors for T2D are integrated to generate a risk score for T2D, proved to be a valuable tool for identifying at-risk subjects [22]. The Finnish Diabetes Risk score (FINDRISK) has been validated in multiple populations, identifies subjects at risk of T2D with a high diagnostic performance by assigning scores for specific categories, including age, BMI, waist circumference, history of antihypertensive drug treatment, high blood glucose, physical activity and daily consumption of fruits, berries, or vegetables [23]. As an example, in 1987 and 1992 FINRISK studies cohorts, a FINDRISK score value ≥ 9 showed, respectively, a diagnostic sensitivity of 0.78 and 0.81, a diagnostic specificity of 0.77 and 0.76, and a positive predictive value of 0.13 and 0.05 [23].

In spite of these tools, however, we are still far from the perfect approach for precisely identifying at-risk individuals and the search for prediction markers of T2D is progressively more needed as the disease prevalence raises.

In this review, we focus on the research efforts made over the past 20 years and address the challenges of the identification of novel biomarkers of T2D risk, moving from the detection of genetic variants in the early 2000s to the discovery of epigenetic changes throughout the last decade. In particular, we will focus on the relevance of DNA methylation biomarkers and critically discuss the current limitations while highlighting the potential value of these novel biomarkers in clinical practice.

## 2. T2D Risk Markers: Genetics

Genetics has long been considered a “fertile ground” for the identification of markers of disease risk; T2D is not an exception [24,25,26]. It is well-documented that some ethnic groups have a higher prevalence of T2D [27,28,29,30]. In the US, Pacific Islanders, South Asians and Filipinos have the highest rates of T2D among all other ethnic groups, including the minorities traditionally considered at high-risk (e.g., Native Americans) [29]. While cultural and environmental factors typical of each ethnicity can explain part of this increased prevalence [12,31], divergent genetic backgrounds are interpreted as a major factor contributing to these differences [12,32]. In addition, the excess risk of T2D is well known to relate to family history of the disease [12,33,34,35,36,37,38,39]. The lifetime risk for first-degree relatives of a patient with T2D is 3–6 times higher than that of age- and weight-matched subjects without a family history of diabetes [36]. In particular, offspring of T2 diabetics have an increased risk ranging from ~ 40 to 70% depending on whether one (with the affected mother conferring the higher risk) or both parents are affected [37]. The relative risk of future disease is ~2–3 in siblings of a patient with T2D and rises to 30 if two siblings have T2D [38]. Furthermore, the concordance of T2D among homozygous twins is ~70% while reaching only 20–30% in heterozygous twins [39].

**Figure 1:**
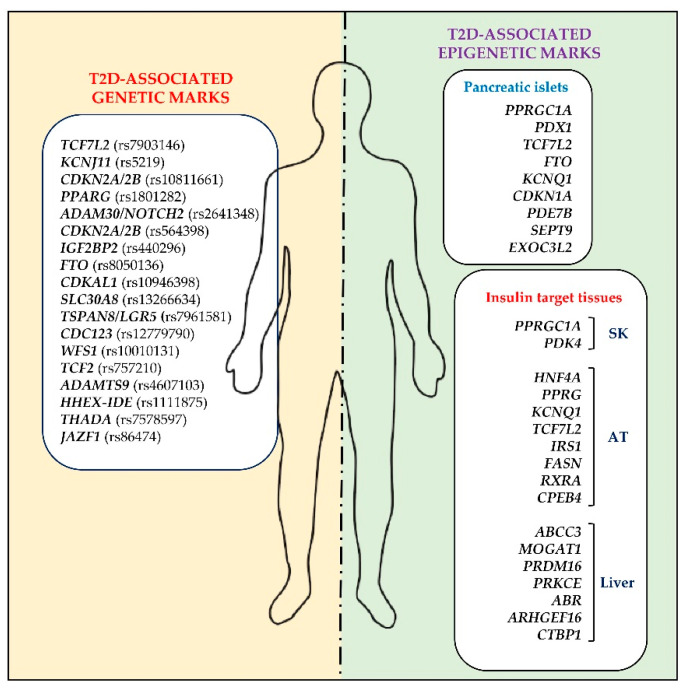
Genetic and epigenetic marks associated with type 2 diabetes. Showing some of the genetic variants (rs or RefSNP) and differentially DNA-methylated genes identified as associated with type 2 diabetes in humans. rs, reference single nucleotide polymorphisms; SK, skeletal muscle; AT, adipose tissue; T2D, type 2 diabetes.

Since early 2000, the combination of different gene discovery approaches, ranging from candidate genes to agnostic analyses, e.g., genome-wide association studies (GWAS), exome and whole-genome sequencing, has led to the identification of over 400 genetic variants associated with T2D or determining quantitative glycemic traits, such as beta-cell function and insulin resistance [24,25,26,40,41]. Some of these genetic variants are reported in Figure 1. The discovery of these variants has had a tremendous impact on identifying the biological mechanisms and novel pathways involved in the pathogenesis of T2D [24,25,26,40,41]. The majority of these variants are located near genes previously unsuspected to play a role in the pathogenesis of T2D or T2D risk, mainly affecting beta-cell function rather than insulin sensitivity in peripheral tissues [42,43,44,45]. Except for a few exon variants that change the amino acid sequence and influence the gene function, such as the p.Pro12Ala of the *peroxisome proliferator-activated receptor-gamma* gene (*PPARG*) or the p.Glu23Lys of the islet *ATP-dependent Kir6.2 potassium channel* gene (*KCNJ11)* [46,47], many are in intronic or regulatory regions or intergenic segments. Interestingly, these findings revealed that non-coding variants might affect human phenotypes [42]. The latter is also the case of the rs7903146 (C/T), located in the intron 4 of the *transcription factor 7-like 2* (*TCF7L2*) gene, which is also the common variant with the strongest association with T2D and with an odds ratio (OR) of 1.35 (T vs. C, 95%CI = 1.31–1.39) [48,49,50]. While the average frequency of a T2D-associated risk allele across populations is 54% [51], the available data from genetic studies have led to the conclusion that the known genetic variants associated with T2D only marginally contribute to disease onset and may, at best, explain up to 15% of T2D heritability, thus generating the missing inheritance issue [44].

From previous studies, we have learned that these variants, alone or in combination, have only small effects on the relative risk of disease. Indeed, except for the rs7903146, no common variant has been found to have significant predictive power for T2D [22,51]. A cross-sectional study of 7232 Finnish men from the Metabolic Syndrome in Men (METSIM) study aimed at identifying previously undiagnosed T2D individuals beyond the FINDRISC investigated the role of biochemical markers and 19 T2D risk polymorphisms and revealed that biochemical markers, but not genetic markers, improve diagnosis [52]. Indeed, the area under the curve (AUC) based on logistic regression models for the identification of undiagnosed T2D subjects with the FINDRISC alone was 0.727 and 0.772 after adding biochemical markers. In contrast, the model did not further improve after adding T2D risk alleles [52]. In addition, an updated analysis of a genetic risk score for 62 single-nucleotide polymorphisms (SNPs) performed in the Framingham Offspring Study generated an AUC for T2D prediction of 0.72 [53]. However, as with the METSIM investigation, adding genetic information caused only a marginal improvement of the AUC for T2D prediction based on clinical factors (AUC for clinical factors, 0.90; AUC for the combined clinical and genetic information, 0.91) [53]. Thus, presently, there is no doubt that common genetic variants do not justify the use of genetic screening to predict T2D. Nevertheless, in the current situation, we may not exclude genetics as a determinant of T2D and as a predictive factor for the disease [54]. The search of risk variants for T2D refers to the “common disease, common variant” hypothesis [55]. GWAS were limited to allelic variants, commonly present in the population (≥5%) [56,57]. Current efforts focus on extending genetic studies to low-frequency (<5%) and rare variants (0.5%) with large effect sizes [58,59]. Manolio et al. estimated that 20 variants with a risk allele frequency of 1% and an OR of 3.0 could account for most familial cases of T2D [59]. A paradigmatic case of the potential predictive power of rare variants is rs61736969 [32]. This non-sense variant (*p.Arg684Ter*) of the *TBC1 domain family member 4* (*TBC1D4*) gene, common only in the Greenlandic Inuit (allele frequency of 14%), explains about 10% of T2D occurrence in Greenland [32]. Homozygous carriers of rs61736969 have markedly elevated 2 h serum insulin levels, post-prandial hyperglycemia, impaired glucose tolerance and a ten-fold higher risk of T2D [32].

More recently, there has been speculation that gene-environment interactions and epigenetic information contribute to the missing inheritance [60,61,62,63,64,65,66]. Furthermore, epigenetics may explain other unsolved issues, including the current epidemiologic changes in T2D or family transmission [60,61,62,63,64]. Further, epigenetic information might be helpful in predicting the risk of T2D [22,67,68,69,70]. These last points will be addressed in the next paragraph.

## 3. DNA Methylation and Type 2 Diabetes

Epigenetics refers to the study of phenotypic changes, eventually heritable, that do not involve alterations in the DNA sequence [71]. It includes several molecular mechanisms determining the epigenetic information (Appendix B) [71]. DNA methylation is the most widely studied mechanism [72,73]. It involves the covalent transfer, catalyzed by the DNA methyltransferases (DNMTs), of a methyl group, to the carbon C5 of cytosine nucleotides to create 5-methylcytosine [72,73]. DNA methylation often occurs in cytosine-guanine dinucleotides (CpG) sites, usually clustered in the CpG islands (CGIs). Cytosine methylation in sites other than CpG sequences has also been described [72,74,75,76]. Quite often, CpG methylation is associated with gene silencing [75,76]. Dense CpG methylation determines stable long-term gene silencing by inhibiting transcription factor binding or recruiting mediators of chromatin remodeling or other gene expression repressors [77]. Conversely, low CpG methylation within gene promoters creates a transcriptionally permissive chromatin state facilitating gene transcription [77]. DNA methylation patterns may also be reversible and change in response to biological, lifestyle, and environmental factors [78,79,80,81,82,83]. Not rarely, specific methylation profiles create molecular abnormalities which cause diseases, including T2D [60,84].

Over the last five years, more than four hundred academic publications have revealed the contribution of CpG methylation to T2D and convincingly associated the changes in DNA methylation profiles to the ongoing epidemics of the disease [60,70]. CpG methylation may reflect the impact of the obesity epidemic on the rise of T2D incidence [60,67]. Moreover, CpG methylation changes can be transmitted across generations and may explain the familial aggregation of T2D, which is not attributable to known genetic variants, often referred to as a missing inheritance [60,63,65,85,86,87]. Within family groups, parents transmit to their offspring not only genes but also lifestyles, eating behaviors and other environmental determinants, which may epigenetically affect gene expression [60,63,65]. Multiple studies have also reported that DNA methylation is among the mechanisms involved in the “developmental origins of health and disease” (DOHaD). This issue addresses how the early life environment impacts the risk of chronic disorders from childhood to adulthood [88,89]. Recent studies in humans reported that changes in the CpG methylation pattern play a role in mediating the association between exposure to prenatal famine and increased risk of obesity, dyslipidemia, T2D and schizophrenia later in life [90]. DNA methylation might also explain unknowns in family transmission among twins [91]. For example, differences in CpG methylation in specific genes have been reported among monozygotic twins discordant for T2D [92,93]. By definition, these subjects share the same genes [94,95]. Finally, CpG methylation has been suggested to be more informative than genetics in predicting T2D in high-risk subjects [96,97]. Some studies linking CpG methylation in pancreatic islets, peripheral tissues or blood to T2D will be deeply discussed in the following sections.

### 3.1. DNA Methylation and Type 2 Diabetes: Pancreatic Islets and Insulin-Target Tissues

Changes in DNA methylation occur in tissues relevant for T2D pathogenesis, including pancreatic islets, skeletal muscle, adipose tissue and liver, contributing to disease onset and evolution.

In 2008, Ling et al. adopted a candidate gene approach to investigate promoter methylation in pancreatic islets from T2D (*n* = 12) and non-diabetic (*n* = 48) multi-organ donors [98]. This study revealed decreased expression of the *PPARG Coactivator 1 Alpha* (*PPARGC1A*) gene and increased DNA methylation within the *PPARGC1A* promoter in islets from T2D donors, both of which correlated with reduced insulin secretion in those subjects [98]. Interestingly, one year later, Barres and coworkers investigated DNA methylation at the *PPARGC1A* gene in skeletal muscle biopsies from a cohort of T2D (*n* = 17) and normal glucose-tolerant (NGT; *n* = 17) male volunteers. These authors reported that hypermethylation of the *PPARGC1A* promoter occurs in T2D patients, which negatively correlates with *PPARGC1A* mRNA expression and is concomitant with reduced mitochondrial content in muscle biopsies of T2D patients [99]. Since then, a number of CpG methylation changes have been reported in pancreatic islets and insulin-target tissues in patients with T2D [94,97,98,99,100,101,102,103,104,105,106,107,108,109]. Most of these changes occurred within genes controlling glucose and lipid metabolism, insulin secretion and function or whole-body energy homeostasis were associated with altered gene expression and shown to functionally affect phenotypes in these tissues, as outlined below (Figure 1) [94,97,98,99,100,101,102,103,104,105,106,107,108,109].

#### 3.1.1. Pancreatic Islets

Immediately after discovering the *PPARGC1A* promoter hypermethylation in T2 diabetic islets, changes of DNA methylation at other genes known to be relevant for β-cell function were also reported [99,100,101,102]. In an initial candidate gene study, Yang et al. compared islets from T2D (*n* = 9) and non-diabetic (*n* = 48) individuals, revealing increased DNA methylation at the *insulin* (*INS*) promoter of four specific CpG sites located 234, 180 and 102 bp upstream and 63 bp downstream of the transcription start site (TSS) [100]. These authors also found that the amount of DNA methylation for CpG −234, −180 and +63 in human pancreatic islets correlated negatively with *insulin* mRNA expression and positively with the level of hemoglobin A1C (HbA1c) [100]. In a second candidate gene study, the same authors showed increased DNA methylation of ten CpG sites in the distal *pancreatic duodenal homeobox 1* (*PDX-1*) promoter and enhancer regions and decreased *PDX-1* gene expression in pancreatic islets from patients with T2D (*n* = 9) compared to non-diabetic donors (*n* = 55) [101]. Later on, thousands of differently CpG methylated loci were identified in human pancreatic islets from T2D and non-diabetic donors by epigenome-wide profiling studies (EWAS) [102,103]. In 2012, Volkmar et al. obtained the first extensive DNA methylation profile in freshly isolated islets from cadaveric human T2D (*n* = 5) and non-diabetic individuals (*n* = 11). These subjects were matched for ethnicity (Caucasian), age and BMI. Two hundred and seventy-six differentially methylated CpGs in T2D pancreatic islets were found, most of which (266 out of 276) encompassed promoter-specific DNA hypomethylation, thereby affecting 254 genes. This study also revealed that prevalent hypomethylation in T2D islets is associated with biological processes involved in adaption to the diabetic environment and pathways implicated in β-cell survival and function [102]. Later on, Dayeh et al. focused on pancreatic islets and analyzed DNA methylation at 479,927 CpG sites along with the transcriptome in T2D (*n* = 15) and non-diabetic (*n* = 35) donors [103]. After correction for multiple testing, these authors identified 1649 CpG sites and 853 genes, including *TCF7L2*, *fat mass and obesity-associated* (*FTO*) and *potassium voltage-gated channel subfamily Q member 1* (*KCNQ1*), with differential DNA methylation in T2D islets. The study also revealed that 102 of the differentially methylated genes, including *cyclin-dependent kinase inhibitor 1A* (*CDKN1A*), *phosphodiesterase 7B* (*PDE7B*), *septin 9* (*SEPT9*) and *exocyst complex component 3 like 2* (*EXOC3L2*), were differentially expressed in T2D islets [103]. Some of the identified genes were also shown to affect, simultaneously, pancreatic β- and α-cell function. For example, *Exoc3l*-silencing reduced exocytosis, while the overexpression of *Cdkn1a*, *Pde7b* and *Sept9* perturbed insulin and glucagon secretion in clonal β- and α-cells, respectively [103].

#### 3.1.2. Insulin-Target Tissues

In 2012, Ribel-Madsen et al. examined global DNA methylation differences in skeletal muscle (*n* = 11 pairs) and subcutaneous adipose tissue (*n* = 5 pairs) from monozygotic twins discordant for T2D [94]. In this study, 789 and 1458 CpG sites were identified, respectively, in skeletal muscle and adipose tissue. Furthermore, methylation changes were validated in the promoters of known T2D-related genes, including *PPARGC1A* in skeletal muscle and *hepatocyte nuclear factor 4 alpha* (*HNF4alpha*) in adipose tissue, both of which exhibited increased methylation in T2D twins [94]. The same year, Kulkarni et al. published results from their investigation of mRNA expression and DNA methylation at genes encoding mitochondrial enzymes in skeletal muscle biopsies from people with normal glucose tolerance (NGT; *n* = 79) or T2D (*n* = 33). In this study, the *pyruvate dehydrogenase kinase 4* (*PDK4*) gene was identified as being associated with T2D [104]. Methylation within the *PDK4* promoter was found to be reduced in T2 diabetics and inversely correlated with *PDK4* expression. Moreover, *PDK4* expression positively correlated with BMI, blood glucose, insulin, C peptide and HbA(1c). Importantly, a 4-month lifestyle intervention program was shown to increase *PDK4* mRNA expression in NGT individuals. Finally, hypomethylation of the *PDK4* promoter in T2Ds coincided with an impaired response of *PDK4* mRNA after exercise [104]. In parallel, Barres and coworkers reported physical exercise-induced dose-dependent expression of *PDK4,* as well as *PPARGC1A* and *peroxisome proliferator-activated receptor delta* (*PPAR-δ*) genes, along with a marked hypomethylation at their promoters in skeletal muscle obtained from healthy sedentary men and women after acute exercise (*n* = 14) [105]. These same authors also demonstrated that promoter methylation of *PPARGC1A* and *PDK4* was altered in skeletal muscle in obese women (*n* = 5) but rescued after Roux-en-Y gastric bypass (RYGB)-induced weight loss [106]. These data provide evidence that exercise, obesity and RYGB-induced weight loss have a dynamic effect on the CpG methylation status of these two T2D target genes.

In the adipose tissue, Nilsson et al. dissected the molecular mechanisms underlying T2D using genome-wide expression and DNA methylation data from monozygotic twin pairs discordant for T2D (*n* = 14) and independent case-control cohorts (cohort 1, *n* = 70 NGT and 50 T2D; cohort 2, *n* = 28 NGT and 28 T2D) [107]. About 15,000 sites, representing 7046 genes and including *PPARG*, *KCNQ1*, *TCF7L2*, and *insulin receptor substrate 1* (*IRS1*), were differentially methylated in adipose tissue from unrelated subjects with T2D, compared with control subjects. Among these, 1410 sites were differentially DNA methylated in the twins who were discordant for T2D [107]. Furthermore, Orozco et al. profiled global methylation levels in subcutaneous abdominal adipose tissue from Finnish people from the METSIM cohort (*n* = 201) and identified 18 high confidence candidate genes. These included known genes, such as the *fatty acid synthase* (*FASN*), *retinoid X receptor alpha* (*RXRA*), *cytoplasmic polyadenylation element-binding protein 4* (*CPEB4*) and novel genes, such as *solute carrier family 1 member 4* (*SLC1A4*), *two-pore segment channel 1* (*TPCN1*) and *strawberry notch homolog 2* (*SBNO2*), associated with diabetes and obesity traits [108]. Using a subset of CpG sites measured in adipose tissue, the authors also developed a DNA methylation-based model to predict the risk of developing T2D [108].

Nilsson et al. examined the global DNA methylation profile in liver biopsies from T2D (*n* = 35) and non-diabetic control (*n* = 60) subjects [95]. They reported 251 individual CpG sites differentially methylated in the livers of T2 diabetics. Some of them are within genes that are potentially relevant to the development of T2D, such as *growth factor receptor-bound protein 10* (*GRB10*), *ATP binding cassette subfamily C member 3* (*ABCC3*), *monoacylglycerol O-acyltransferase 1* (*MOGAT1*) and *PR/SET domain 16* (*PRDM16*). Furthermore, 29 other genes, including the long coding RNA *H19*, displayed differential DNA methylation and gene expression in human T2D livers, supporting a functional role of these epigenetic changes in the liver dysfunction occurring in T2D [95]. Kirchner et al., searching for aberrant pathways underlying the development of insulin resistance, investigated the DNA methylome and transcriptome in the liver from severely obese men with (*n* = 8) or without (*n* = 7) T2D and in non-obese control subjects (*n* = 7) [109]. Among genes with altered expression and DNA-methylation changes in obese T2D individuals compared to non-obese controls, the *protein kinase C epsilon* (*PRKCE*), *active BCR-related* (*ABR*)*,* and *rho guanine nucleotide exchange factor* (*ARHGEF16*) belonging to the nerve growth factor signaling and the *C-terminal binding protein 1 (CTBP1*), *cyclin D1* (*CCND1*) and *wingless-type MMTV integration site family, member 11* (*WNT11*) belonging to the Wnt signaling pathways emerged [109]. Moreover, very recently, Krause et al. reported the downregulation of *IRS2* expression in the liver of obese individuals with T2D (*n* = 31) compared to obese individuals without T2D (*n* = 50) by adopting a candidate gene approach through a multi-layered epigenetic mechanism [110]. The author suggested that this regulation is facilitated by the variability in hepatic *IRS2* DNA methylation within transcription-factor binding motifs and the upregulation of hepatic miRNA *let-7e-5p* in T2 diabetics [110]. The studies reported in Section 3.1.1 and Section 3.1.2 are summarized in Table 1.

### 3.2. DNA Methylation and Type 2 Diabetes: Blood Cells

Blood is a readily accessible tissue for biomarker identification [111,112]. Recent evidence suggests that blood-derived DNA methylation changes may mirror the epigenetic variation occurring of metabolically relevant dysfunctional tissues [68,113,114] and also represent valid tools for discovering DNA methylation marks associated with T2D [96,97,114,115,116,117,118,119,120,121].

In 2012, Toperoff et al. adopted a multi-step study strategy including a cross-sectional case-control (first arm) and a further longitudinal retrospective (second arm) analysis to explore the hypothesis that DNA methylation variation in human peripheral blood predisposes T2D susceptibility [97]. In this genome-scale screening study, including 1169 Israeli residents of Jewish origin (cases, *n* = 710 and controls, *n* = 459), the authors revealed an increased number of differentially methylated regions (DMR) at genomic loci previously associated with T2D. The study also revealed a 3.35% hypomethylation at a CpG site, located within the intron 1 and 11 bp upstream of the obesity and T2D-associated rs1121980 of the *FTO* gene, in T2 diabetics (*n* = 198) relative to non-diabetics (*n* = 233). This specific hypomethylated site was independent of the rs1121980 (A = risk allele) and persisted among individuals carrying the sequence-risk alleles. In an independent cohort from the Jerusalem LRC longitudinal study, authors found significant hypomethylation at this same CpG site in young individuals, which subsequently progressed to T2D (*n* = 58) compared to those who remained healthy (*n* = 64), indicating that DNA methylation variation at this specific genomic position is an early marker of T2D. This investigation further demonstrated that (i) for each 1% decrease in CpG methylation at this site, the OR of being included in the T2D group increases by 6.1% and (ii) based on the receiver-operating characteristic area under the curve (ROC AUC) comparison, the CpG site methylation at the *FTO* gene is more effective in detecting T2D risk (ROC AUC = 0.638) than either the identification of rs7901695 at the *TCF7L2* gene (ROC AUC = 0.55) or that of the combination of the 18 best established genetic variants (ROC AUC = 0.6) [97].

Several other studies have later analyzed DNA methylation in blood cells, searching for new biomarkers that can improve the prediction of T2D [114,115,116,117,118,119,120,121]. In 2014, Canivell et al. used a candidate gene approach and compared peripheral blood DNA methylation profiles within the promoter of the *TCF7L2* risk gene in newly diagnosed, drug-naïve T2D patients (*n* = 93) and age- and BMI-matched controls (*n* = 93). This paper reported that 59% of the CpG sites analyzed in the *TCF7L2* promoter had significant differences in T2D patients and matched controls [115]. Furthermore, the methylation of specific CpG sites on the *TCF7L2* promoter in blood correlated with fasting glucose, total cholesterol and low-density lipoprotein cholesterol, shedding new light on the interplay between epigenetics and the *TCF7L2* diabetes susceptibility gene in the development of T2D [115].

Yuan et al. performed an integrated epigenomic analysis in whole-blood DNA from participants of the Twins UK cohort. This study included pairs of monozygotic twins (pairs of T2D-discordant twins, *n* = 17, pairs of T2D concordant twins *n* = 3 and pairs of healthy concordant twins *n* = 7), but also unrelated T2D cases (*n* = 42) and non-diabetic controls (*n* = 221) matched for age, BMI and sex [116]. In particular, the authors reported predominant hypermethylation of the DMR. Moreover, they identified the strongest T2D-related hypermethylated signal (chr18:56336501-56337000) in a region located at 2kb upstream of the TSS within the 5′ promoter of the *mucosa-associated lymphoid tissue lymphoma translocation protein 1* (*MALT1*) gene, involved in antigen receptor-mediated lymphocyte activation, in the development and function of B and T cells, and in insulin and glycemic pathways [116].

In 2015, Chambers et al. performed a nested-control study in Indian Asians (*n* = 2664) from the 8-year follow-up participants in the London Life Sciences Prospective Population (LOLIPOP) study, and Europeans from the LOLIPOP study (*n* = 1141) and the KORA S3 and S4 studies (*n* = 382). This work identified peripheral blood methylation markers associated with incident T2D, five loci near the *ATP-binding cassette sub-family G member* 1 (*ABCG1*), *sterol regulatory element-binding protein 1* (*SREBF1*), *phosphoethanolamine/phosphocholine phosphatase* (*PHOSPHO1*), *suppressor of cytokine signaling 3* (*SOCS3*) and *thioredoxin-interacting protein* (*TXNIP*) genes [117]. The relative risk of developing T2D per 1% increase in methylation at these marks was 1.09 for the cg06500161 (*ABCG1*) and 1.07 for the cg11024682 (*SREBF1*) but decreased by 6% for both the cg02650017 (*PHOSPHO1*) and the cg18181703 (*SOCS3*) and by 8% for the cg19693031 (*TXNIP*) [117]. Furthermore, the DNA methylation score, obtained by combining results for the five loci, was associated with a four-fold higher risk of future T2D incidence in the upper versus the lower relative risk quartiles of the DNA methylation score and was independent of established risk factors for T2D [117]. Besides, this study revealed that the methylation patterns differ among Indian Asians and Europeans and, in Indian Asians, are associated with increased risk of developing T2D, suggesting that the assessment of DNA methylation might help in predicting the magnitude of T2D risk [117].

The same year, Kulkarni et al. examined the association of DNA methylation in peripheral blood cells and T2D, fasting blood glucose and homeostatic model assessment of insulin resistance (HOMA-IR) in 850 pedigreed Mexican-Americans, 21% of whom had T2D and 17% impaired fasting glucose [118]. In this study, five CpG sites that independently explained 7.8% of the heritability of T2D emerged. Interestingly, two of these sites were the previously reported cg19693031 at *TXNIP* and the cg06500161 at *ABCG1* (see above). In this study, the former site was further shown to be strongly associated with both fasting blood glucose and HOMA-IR, while the latter with HOMA-IR alone [118].

An independent study, published in 2016, was performed with participants to the Botnia studies and aimed at examining DNA methylation at the *ABCG1*, *PHOSPHO1*, *SOCS3*, *SREBF1* and *TXNIP* loci [96]. These individuals were subjected to a mean 8-year follow-up, were non-diabetic at the inception of the study and either maintained normal glucose tolerance (*n* = 129) or developed diabetes (*n* = 129). In these individuals, only DNA methylation at the loci within cg06500161 (*ABCG1*) and cg02650017 (*PHOSPHO1*), but not at cg18181703 (*SOCS3*), cg11024682 (*SREBF1*) and cg19693031 (*TXNIP*), in blood DNA was related to the risk of T2D in subsequent years [96]. In particular, DNA methylation at the *ABCG1* locus in blood cell DNA of healthy individuals (baseline) was associated with a 9% increased risk of subsequent T2D and positively correlated with BMI, HbA1c, fasting insulin and triglyceride levels. DNA methylation at the *PHOSPHO1* locus in blood cell DNA was associated with a 15% decreased risk of future T2D and positively correlated with high-density lipoprotein (HDL) levels. In the same study, with an independent population of monozygotic twin pairs (*n* = 17), Dayeh et al. reported evidence that blood cell DNA methylation at these two loci reflects alterations in target tissues of major relevance in T2D development. Indeed, CpG methylation at the *ABCG1* locus was increased in the adipose tissue as well as in the blood of diabetic twins but not in the discordant twin who had no T2D. Furthermore, DNA methylation at the *PHOSPHO1* locus was decreased in skeletal muscle from the diabetic compared to non-diabetic twins [96].

In 2017, Walaszczyk et al. published a replication study for CpGs robustly associated with T2D in individuals living in the Netherlands, initially recruited in the Lifelines study (T2D cases, *n* = 100; control subjects *n* = 100) [119]. In this work, 5 out of the 52 identified CpGs, the cg06500161 (*ABCG1*), cg24531955 (*Lysyl Oxidase Like 2*, *LOXL2*), the cg19693031 (*TXNIP*), the cg02711608 (*solute carrier family 1 member 5*, *SLC1A5*) and the cg11024682 (*SREBF1*) were replicated in the blood cells and thus nominally associated with T2D. In particular, loci at the *ABCG1* and *SREBF1* genes were found hypermethylated, while loci at *TXNIP*, *LOXL2* and *SLC1A5* were hypomethylated in the T2D compared with control individuals [119].

A case-control study focusing on novel methylation-variable positions associated with T2D has been subsequently published by Cardona et al. [120]. In this effort, three different populations were investigated. A group of English and Welsh individuals with (*n* = 563) or without (*n* = 701) incident T2D from the population-based European Prospective Investigation into Cancer and Nutrition (EPIC)-Norfolk cohort were followed for up to 11-years before T2D onset and subjected to EWAS. Then, two further replication cohorts were studied, represented by 1074 cases and 1590 controls from the LOLIPOP study and by 403 cases and 2204 controls from the offspring cohort of the Framingham Heart study (FHS). In this effort, the authors identified 15 novel methylation variable positions (MVPs) with robust associations with incident T2D and further validated by three MVPs previously identified near the *TXNIP* (cg19693031), *ABCG1* (cg06500161) and *SREBF1* (cg11024682) genes. The identified signals were attenuated by adjustment for differences in BMI and glycemia developed before baseline recruitment. Nominal associations of DNA methylation intensity of the cg06500161 at *ABCG1* in blood cells and adipose tissue and the cg19693031 at *TXNIP* in blood and skeletal muscle were also reported, along with evidence of positive correlation of DNA methylation between blood cells, liver, pancreas, adipose tissue and skeletal muscle for 12 of the 18 identified MVPs. Finally, in this same study, evidence for a direct causal association with T2D was obtained for the cg00574958 at the *carnitine palmitoyl transferase I* (*CPT1A*) gene.

Krause et al. tested the methylation loci cg06500161 (*ABCG1*) and cg11024682 (*SREBF1*) as classifiers for diabetes stratification in two Northern German cohorts [121]. One population was represented by healthy subjects without T2D (*n* = 176), where DNA methylation was investigated in blood samples. The second one was of obese subjects with or without overt T2D (*n* = 100), where DNA methylation was measured in liver and adipose tissue [121]. In the case of cg06500161, the authors reported that blood CpG methylation at the *ABCG1* locus correlated with glucose levels and HOMA-IR. Also, this locus was influenced by the adjacent SNP rs9982016. In the case of cg11024682, methylation at the *SREBF1* locus correlated with glucose levels, while both blood and liver CpG methylation negatively correlated with BMI. Likewise, it was shown that a methylation risk score based on blood DNA methylation at cg06500161 and cg11024682 enables stratification of the cohorts into insulin-resistant and insulin-sensitive or lean and obese subjects [121], indicating that DNA methylation in blood cells is a valid approach for stratification of risk groups and may be used for T2D risk prediction.

In 2020, García-Calzón et al. investigated the hypothesis that blood-based epigenetic markers may discriminate response and tolerance to metformin therapy in T2D patients [122]. In this effort, genome-wide DNA methylation was analyzed in three different cohorts of drug-naïve patients with T2D at the time of diagnosis. Swedish individuals from the All New Diabetics In Scania (ANDIS) study (*n* = 47 responders/non-responders and *n* = 83 tolerant/intolerant to metformin therapy) made the discovery cohort. Additional T2 diabetics from the ANDIS study (*n* = 87 responders/non-responders and *n* = 48 tolerant/intolerant to metformin therapy) constituted the first replication cohort. In contrast, two other groups of European subjects represented by Swedish and Latvian T2 diabetics (*n* = 78 responders/non-responders and *n* = 20 tolerant/intolerant to metformin therapy) from the All New Diabetics in Uppsala County (ANDiU) and the Optimized program of personalized treatment of type 2 diabetes (OPTIMED) cohorts, respectively, were included in the second replication cohort. In a combined meta-analysis of discovery and replication data, the authors reported that 11 sites, including the cg01070242 (*Septin 11*, *SEPT11*) and cg07511259 (*Cystatin SN*, *CST1*), were differently methylated between glycemic responders and non-responders to metformin therapy; while other four CpG sites, including the cg12356107 (*Forkhead Box A2*, *FOXA2*) and cg02994863 (*Phosphoglucomutase 1*, *PGM1*), resulted differentially methylated between metformin tolerant and intolerant patients. Additionally, it was shown that methylation at these sites discriminates between glycaemic responders/non-responders and participants tolerant/intolerant to metformin therapy. Indeed, a combined weighted methylation risk score (MRSs) based on the 11 CpG sites identified in the metformin response arm of the study sorted out between glycemic responders and non-responders to metformin with ROC AUCs ranging between 0.80 to 0.98. Furthermore, the MRSs of the four CpG sites found in the metformin intolerance arm gave adequate separation between metformin-intolerant and metformin-tolerant participants with ROC AUCs ranging between 0.85 to 0.93. Should these results be confirmed in other T2D populations, the use of these blood-based epigenetic markers may be considered to help identify T2D patients who should receive metformin. Interestingly, two years before, Karaglani and coworkers studied pharmaco-epigenetic correlations among promoter methylation at *KCNJ11* and *ATP binding cassette subfamily C member 8* (*ABCC8*) genes and mild hypoglycaemic events in Greek T2D subjects under sulfonylurea treatment (*n* = 88, who experienced, and *n* = 83, who had never experienced hypoglycemia) and demonstrated for the first time that CpG methylation at *ABCC8* was associated with non-hypoglycemic events in sulfonylureas-treated T2D patients [123]. Overall, these two studies support the further development of blood-based DNA methylation markers to help clinical decision-making for the treatment of T2 diabetics.

Finally, most recently, Juvinao Quintero et al. carried out a large meta-analysis of independent EWAS, investigating changes of blood DNA methylation in prevalent T2Ds, in four European studies [124]. These included the Avon Longitudinal Study of Parents and Children (ALSPAC), the Lothian Birth Cohort of 1936 (LBC1936), and two sub-cohorts of the Rotterdam study, RSIII-1 and RS-Bios [124,125,126,127,128,129,130,131]. Collectively, this meta-EWAS included methylation data from 3428 subjects (cases; *n* = 340) and revealed that T2D is strongly associated with DNA methylation at six specific CpGs. Three of them were the loci at the *TXNIP* (cg19693031), *ABCG1* (cg06500161) and *CPT1A* (cg00574958) genes already identified in previous studies. The other three were newly identified CpGs associated with prevalent T2D in Europeans. These CpGs mapped close to the *Histone deacetylase 4* (*HDAC4*; cg00144180), *Synemin* (*SYNM*; cg16765088) and *hsa-miR23a* (*MIR23A*; cg24704287) genes. Using the ALSPAC population, the authors also reported that the combination of the six differentially methylated CpG sites accounted for 11% of the total variation in T2D, which was very similar to the variation accounted for by the model for common risk factors (age, sex, BMI and smoking). Moreover, in this population, the variation attributed to these common risk factors and methylation at the six CpG sites was 22% and adding the fasting glucose to this predictive model captured 68% of the variation in T2D [124]. Whether and how these findings can be generalized in non-European populations and the potential roles of these epigenetic markers in T2D etiology or in determining long-term consequences of T2D remains to be established.

The blood-based DNA methylation marks associated with T2D and the studies reported in this section are summarized in Table 2.

## 4. DNA Methylation in Clinical Practice: A Biomarker for T2D?

Biomarker discovery from translational epigenetics is necessary for improving the detection of individuals at high risk of T2D and for improving diagnostic and prognostic strategies, as well as for predicting responses to therapy and lifestyle interventions. However, the identification of novel biomarkers and the use of those already available is still embryonic and improvements in the range and quality of these tools are still needed. To date, translational epigenetics has been mainly applied to the Oncology field [132]. Tests for the screening of early colorectal cancer (CRC) in blood or stool specimens are among the few already approved by the US Food and Drug Administration (FDA) and European Conformity of in vitro diagnostic medical devices (CE-IVD) [133,134,135,136]. These tests detect CpG methylation at specific gene promoters, including the *bone morphogenetic protein 3* (*BMP3*) and *NDRG family member 4* (*NDRG4*) genes in stool [133] and *SEPT9* gene in peripheral blood samples [134,135]. In particular, the *SEPT9* methylation test stands out for its high diagnostic sensitivity (75–81%) and specificity (96–99%) for CRC. More recently, reduced DNA methylation and increased gene expression of the *SEPT9* gene have been reported in pancreatic islets of T2D individuals [103]. However, whether this test might be used as a diagnostic tool for T2D needs to be explored.

In the field of metabolism, there are few clinical trials currently in progress focusing on the clinical application of epigenetic marks in monitoring the evolution of diabetes and lifestyle intervention [137,138,139]. One of these studies (ClinicalTrials.gov Identifier: NCT02982408) aims at evaluating the impact of overfeeding and exercise training in individuals with and without increased risk of T2D, having as major endpoint the investigations of epigenetic marks in adipose tissue and in skeletal muscle at baseline and after lifestyle interventions [137]. A further study (NCT01911104) is investigating exercise resistance in T2 diabetics and healthy participants. One of the endpoints of this effort is the evaluation of the promoter methylation status of genes involved in fuel metabolism and known to be activated by exercise in skeletal muscle tissue [138]. An independent investigation aims at understanding the role of DNA methylation in insulin resistance in skeletal muscle and blood cells from metabolically well-characterized healthy, obese, non-diabetic and type 2 diabetic volunteers. In particular, the objective of this clinical study is to define CpG methylation patterns within the promoter of *PPARGC1A* and other genes involved in mitochondrial biogenesis, oxidative phosphorylation, extracellular matrix and cytoskeleton proteins in insulin resistance in response to an acute episode of exercise, and upon eight weeks of a training exercise [139].

Altogether, these studies revealed specific methylation patterns associated with T2D and detectable blood and pancreatic beta-cells, liver, skeletal muscle and adipose tissue [94,95,96,97,98,99,100,101,102,103,104,105,106,107,108,109,110,116,117,118,119,120,121,124]. Some appear before the T2D debut, feature a high effect size on disease risk and show significant potential as disease markers [94,95,96,97,98,99,100,101,102,103,104,105,106,107,108,109,110,116,117,118,119,120,121,124]. However, most of the studies currently available cannot be considered definitive, making the routine use of these markers in T2D clinical practice not yet feasible. The approaches adopted in these studies to quantify DNA methylation are heterogeneous. In some, analysis of CpG methylation within genes associated with T2D has been used. Others focused on unbiased global DNA methylation [94,95,96,97,98,99,100,101,102,103,104,105,106,107,108,109,110,116,117,118,119,120,121,124]. Candidate gene analyses took advantage of methodologies including methylated DNA immunoprecipitation (MeDIP), methylation-specific PCR and bisulfite pyrosequencing [96,98,99,100,101,104,105,110,115]. Unbiased and EWAS approaches used microarray-based methylation assays, bead chip arrays, or MeDIP sequencing [94,95,97,102,103,106,107,108,109,116,117,118,119,120,121,124]. This variability between different studies renders result comparison more difficult. However, epigenetics is expanding at such a rapid rate, and new, more accurate methods are being developed every day so that interpretation is expected to become more conclusive.

The cross-sectional, case-control design of most of these studies causes further limitations. The majority of them were performed in a population of unrelated T2D and healthy subjects, or homozygous twin pairs discordant for T2D or both [94,95,96,97,98,99,100,101,102,103,104,107,108,109,110,116,117,118,119,120,121,124]. Thus, they were unable to clarify whether DNA methylation changes precede T2D onset. Capturing the dynamics of epigenetic changes at multiple time points during life is important in searching for disease risk biomarkers. To achieve this goal, longitudinal studies in large cohorts of healthy individuals that follow up (to track T2D onset) are necessary. However, with few exceptions [96,97,108,117,120], the higher costs and the time requirement of this strategy strongly reduce its feasibility.

Additional limitations affecting data interpretation originate from the incomplete classification of subjects in terms of demographics, lifestyle, clinical history and lack of adjustment for confounding factors, including gender, age, BMI, glycemic, as well as other metabolic relevant traits or chronic medications [119].

The ethnicity issue is crucial for data interpretation, as different patterns of DNA methylation have been found in different ethnic groups. Moreover, markers identified in a specific population may not be validated in another. In part, these apparent discrepancies are explained by different genetic ancestry and lifestyle, cultural and environmental factors, which are divergent among different ethnicities or countries. Nonetheless, changes in DNA methylation at some loci were confirmed in T2 diabetic individuals with diverse ethnic backgrounds, as in the case of the *ABCG1* and *TXNIP* loci [117,118,119,140,141,142].

Cytotype composition also influences the outcome of analytic procedures as methylome profiles may vary between different cell types [143,144,145]. The greater the cell heterogeneity within tissue samples, the greater the complexity in interpreting DNA methylome analysis [144,145]. Blood samples, for example, contain many different leukocyte subpopulations, which may exhibit unequal distribution among different individuals [146]. Several methods have been developed to avoid such potential confounders, such as adjustment for directly measured cell count or reference-based cell count, as with the Houseman method [147,148,149].

Finally, the DNA methylation signature in blood-borne DNA does not necessarily reflect CpG methylation in other tissues [119,120], which may limit the use of DNA methylation biomarkers for T2D, which have been identified in blood [96,121]. Ideal biomarkers should be detectable in easily accessible samples, such as blood, and reflect changes in less accessible tissues [130,150,151,152]. In subjects with T2D, a variety of DNA methylation changes have been reported in tissues important in the pathogenesis of T2D, such as pancreatic beta-cells, liver, skeletal muscle or adipose tissue [97,98,99,100,101,102,103,104,105,106,107,108,109,110,111,112,113]. These changes do not always occur in blood cells, indicating that they may be tissue-specific [96,117,119]. With other CpG methylation changes, however, replication in blood and other cell types is extensive [96], supporting an even greater informativity as T2D biomarkers.

## 5. Conclusions

Characterization of DNA methylome in T2 diabetics has been made possible by integrating large amounts of data from site-specific CpG methylation identified in candidate genes and even more powerful epigenome-wide studies [94,95,96,97,98,99,100,101,102,103,104,105,106,107,108,109,110,116,117,118,119,120,121,124]. These efforts have provided significant amounts of information on gene function in tissues, explaining endophenotypic differences among T2 diabetics and healthy individuals [94,95,96,97,98,99,100,101,102,103,104,105,106,107,108,109,110]. Furthermore, variations in DNA methylation profiles could be associated with lifestyle factors, in particular to physical exercise and overfeeding [153,154,155,156], suggesting that these modifications may capture signals from the environment and mediate the progression from health to disease.

The identification of specific CpG methylation marks in blood cell DNA from T2D subjects is still in a developmental stage at present. Nevertheless, the significant research efforts devoted to this objective over the past ten years hold convincing promise to convert current advances in this area of epigenetics into novel and easily detectable biomarkers for assessment of risk and progression towards T2D in patients [96,116,117,118,119,120,121,124]. Some of the blood DNA methylation marks identified in T2 diabetics already appear to be clinically relevant [96,117,119]. They have been replicated in tissues involved in insulin secretion or responsiveness, implying translation of epigenetic information from metabolically relevant tissues to blood [96,117,119]. Moreover, the contribution of DNA methylation marks to the risk of developing T2D in individuals is likely to be much larger than that of genetic marks [96,117,119], as the genetic risk of T2D is dependent on the presence of risk alleles [12]. Conversely, CpG methylation changes are more dynamic, and the relative risk to belong to the T2D versus the non-diabetic group increases for every 1% change [96,97,117,119], so that even small variations of CpG methylation may cause a consistent increase of T2D risk. In the future, methylation biomarkers in blood-borne DNA are also expected to complement currently available T2D biomarkers, such as plasma glucose and HbA1c [124]. These biomarkers are only useful after the disease onset. In addition, CpG methylation markers may represent a powerful tool for the early detection of T2D complications [68,69] and for the stratification of drug-naïve patients with T2D in responders/non-responders to glycemic-lowering mediations [122,123].

Search for epigenetic markers also include miRNAs and histone modifications [132]. Emerging evidence in blood samples of T2D individuals revealed their potential use as disease biomarkers [157,158,159], but this further epigenetic information was not within the scope of this review. In addition, other molecules are emerging as relevant candidates associated with T2D and its complications beyond the epigenetic marks [157,158,159]. These include acute-phase proteins (e.g., C-reactive protein and fibrinogen) and pro-inflammatory cytokines (e.g., interleukin 6 and tumor necrosis factor-alpha) [159,160,161]. Blood netrin-1, a laminin-related protein, is also increased in individuals with pre-diabetes and with T2D [159,162]. Whether these latter molecules may serve as markers remains to be firmly established.

The generation of multi-marker models by the data integration of epigenetic markers with other emerging factors will be essential for improving individual risk assessment models for T2D. Finally, it has to be reported that the current methods used in the majority of the methylome studies to which this review has been dedicated only cover a modest part of the DNA methylome (∼1.5%) [67]. A complete dissection of CpG methylation, which will likely be completed in the next few years, will generate a more complete picture of the epigenome in humans and diseases like T2D, enabling the identification of additional biomarkers with greater predictive capacity for T2D and potentially useful for prediction of T2D-related complications and assignment of more adequate treatment to each T2D patients.

## Figures and Tables

**Table 1:** Methylation studies in pancreatic islets and insulin-target tissues.

Research Article	Study Information	Participants	Tissues Relevant for T2D Pathogenesis	Main Findings
Ling et al. (2008) [98]	Candidate gene (bisulfite sequencing)	T2 diabetic (*n* = 12) and non-diabetic (*n* = 48) multi-organ donors	Pancreatic islets	Two-fold increase in DNA methylation of *PPARGC1A* promoter (−986/−746 bp from TSS) in T2 diabetic pancreatic islets
Barres et al. (2009) [99]	Candidate gene (MeDIP assay; bisulfite sequencing)	T2 diabetic (*n* = 17), impaired glucose-tolerant (IGT; *n* = 8) and normal glucose tolerant (NGT; *n* = 17) male volunteers	Skeletal muscle	The highest proportion of DNA methylation of *PPARGC1A* (−337/−37 bp from TSS) within non-CpG nucleotides in T2 diabetic skeletal muscle
Yang et al. (2011) [100]	Candidate gene (MALDI-TOF mass spectrometry-based bisulfite sequencing)	T2 diabetic (*n* = 9) and non-diabetic (*n* = 48) deceased donors	Pancreatic islets	Increased DNA methylation in 4 CpGs of *INS* gene (−234, −180, −102 and +63 bp from TSS) in T2 diabetic pancreatic islets
Barres et al. (2012) [105]	Global DNA methylation of candidate genes (MeDIP assay and bisulfite sequencing)	Sedentary cohort under an acute bout of exercise (*n* = 14)	Skeletal muscle	Marked hypomethylation of *PPARGC1A*, *PDK4*, and *PPAR-δ* promoter and dose-dependent increase of *PPARGC1A* (−2337/−139 bp from TSS), *PDK4*, and *PPAR-δ* mRNA expression in skeletal muscle from participant under exercise program
Kulkarni et al. (2012) [104]	Candidate gene (bisulfite sequencing)	T2 diabetic (*n* = 33) and normal glucose tolerant (NGT; *n* = 79) volunteers randomized to 4-month lifestyle intervention	Skeletal muscle	Reduced DNA methylation of the *PDK4* promoter (+160/+446 bp from TSS) and increased *PDK4* mRNA expression in T2 diabetic skeletal muscle
Ribel-Madsen et al. (2012) [94]	^a^ EWAS (methylation bead chip) ^b^ Genomic region validation (bisulfite sequencing)	Monozygotic twins discordant for T2D (SK, *n* = 11 pairs; SAT, *n* = 5 pairs)	Skeletal muscle and adipose tissue	Increased DNA methylation in the promoter of *PPARGC1A* and *HNF4alpha* genes in T2 diabetic SK and SAT, respectively
Volkmar et al. (2012) [102]	EWAS (methylation bead chip)	T2 diabetic (*n* = 5) and non-diabetic (*n* = 11) deceased donors	Pancreatic islets	276 CpGs differentially methylated in T2 diabetic pancreatic islets (mostly encompassing promoter-specific DNA hypomethylation)
Yang et al. (2012) [101]	Candidate gene (MALDI-TOF mass spectrometry-based bisulfite sequencing; bisulfite pyrosequencing)	T2 diabetic (*n* = 9) and non-diabetic (*n* = 55) deceased donors	Pancreatic islets	Increased DNA methylation in 10 CpG sites in the distal *PDX-1* promoter and enhancer regions (−3767/−27 from TSS) in T2 diabetic pancreatic islets
Barres et al. (2013) [106]	^a^ Global CpG and non-CpG methylation (luminometric methylation assay) ^b^ Candidate gene (bisulfite sequencing)	Normal-weight women (*n* = 6), obese women pre-RYGB (*n* = 5) and obese women post-RYGB (*n* = 5)	Skeletal muscle	Altered DNA methylation of *PPARGC1A* and *PDK4* promoter in obese woman skeletal muscle; restored DNA methylation of these promoters to non-obese levels after RYGB-induced weight loss
Dayeh et al. (2014) [103]	^a^ EWAS (methylation bead chip) ^b^ Genomic region validation (bisulfite pyrosequencing)	T2 diabetic (*n* = 15) and non-diabetic (*n* = 34) deceased donors	Pancreatic islets	1649 CpGs, including *TCF7L2*, *FTO*, *KCNQ1*, *CDKN1A*, *PDE7B*, *SEPT9*, and *EXOC3L2*, differentially methylated in T2 diabetic pancreatic islets
Nilsson et al. (2014) [107]	EWAS (methylation bead chip)	Monozygotic twins discordant for T2D (*n* = 14 pairs) and T2 diabetics (Cohort 1, *n* = 50; Cohort 2, *n* = 28) and normal glucose tolerant subjects (NGT; Cohort 1, *n* = 70; Cohort 2, *n* = 28)	Adipose tissue	Differential DNA methylation of 7046 genes, including *PPARG*, *KCNQ1*, *TCF7L2*, and *IRS1*, in adipose tissue from unrelated subjects with T2D
Nilsson et al. (2015) [95]	EWAS (methylation bead chip)	T2 diabetic (*n* = 35) and non-diabetic (*n* = 60) donors undergone RYGB	Liver	251 CpGs, including *GRB10*, *ABCC3*, *MOGAT1*, *PRDM16*, and the long coding RNA *H19*, differentially methylated in T2 diabetic liver
Kirchner et al. (2016) [109]	^a^ EWAS (methylation bead chip)) ^b^ Genomic region validation(bisulfite pyrosequencing)	Randomly chosen subjects (non-obese, *n* = 7; obese non-diabetic, *n* = 7; and obese T2 diabetic, *n* = 8)	Liver	Altered CpG methylation and mRNA expression of genes belonging to the nerve growth factor signaling (*PRKCE*, *ABR,* and *ARHGEF16*) and the Wnt signaling (*CTBP1*, *CCND1*, and *WNT11*) in obese T2 diabetic liver
Orozco et al. (2018) [108]	EWAS (*RRBS*-seq)	Individuals from the METSIM cohort (*n* = 201)	Adipose tissue	DNA methylation at *FASN*, *RXRA*, *CPEB4*, *SLC1A4*, *TPCN1*, and *SBNO2* genes associated with diabetes and obesity traits metabolic traits; development of a DNA methylation-based model to assess T2D risk
Krause et al. (2020) [110]	Candidate gene (bisulfite pyrosequencing)	Obese individuals with (*n* = 31) or without T2D (*n* = 50)	Liver	Multi-layered epigenetic regulation of *IRS2* expression (high variability of *IRS2* DNA methylation within transcription-factor binding motifs and increased miRNA let-7e-5p) in obese T2 diabetic liver

Abbreviations: ^a^, phase 1 of the study design; ^b^, phase 2 of the study design; MALDI-TOF, matrix-assisted laser desorption/ionization-time-of-flight; MeDIP, methylated DNA immunoprecipitation; EWAS, epigenome-wide association study; SK, skeletal muscle; SAT, subcutaneous adipose tissue; RYGB, roux-en-Y gastric bypass; METSIM, metabolic syndrome in men.

**Table 2:** Methylation Studies in blood.

Research Article	Study Information	Cohorts—c/c	Gene (CG Site)	Position	T2D Risk/Main Findings	
Chambers et al. (2015) [117]	^a^ Nested case-control study EWAS (methylation bead chip); ^b^ Replication testing candidate (bisulfite pyrosequencing)	^a^ Indian Asians from the LOLIPOP study, (1074/1590); ^b^ Europeans from the LOLIPOP study, KORA S3, and KORA S4 studies [306/6760] ^§^	*ABCG1* (cg0650016)	Body	RR (95%CI)	1.09 (1.07–1.11)
			*PHOSPHO1* (cg02650017)	Body		0.94 (0.92–0.95)
			*SOCS3* (cg18181703)	Body		0.94 (0.92–0.96)
			*SREBF1* (cg11024682)	Body		1.07 (1.04–1.09)
			*TXNIP* (cg19693031)	3′-UTR		0.92 (0.90–0.94)
Kulkarni et al. (2015) [118]	Family-based study EWAS (methylation bead chip)	Mexican-American from the San Antonio Family Heart Study [850 (~21% T2D)]	*ABCG1* (cg06500161)	Body	Association between T2D status and methylation levels	
			*TXNIP* (cg19693031)	3′-UTR		
			*SAMD12* (cg01192487)	5′-UTR		
Dayeh et al. (2016) [96]	Replication testing candidate (bisulfite pyrosequencing)	Europeans from the Botnia prospective study (129/129) ^§^	*ABCG1* (cg0650016)	Body	RR (95%CI)	1.09 (1.02–1.16)
			*PHOSPHO1* (cg02650017)	Body		0.85 (0.75–0.95)
Walaszczyk et al. (2017) [119]	Replication testing EWAS (methylation bead chip)	Europeans from the Lifelines study (100/98)	*ABCG1* (cg06500161)	Body	Association between T2D status and methylation levels	
			*SREBF1* (cg11024682)	Body		
			*TXNIP* (cg19693031)	3′-UTR		
			*LOXL2* (cg24531955)	3′-UTR		
			*SLC1A5* (cg02711608)	1st Exon		
Karaglani et al. (2018) [123]	Case-control study (MeDIP on candidate genomic regions)	^a^ Europeans with T2D under sulfonylureas treatment who experienced hypoglycemic events (88/83)	*ABCC8* (/)	Promoter	Association of DNA methylation at *ABCC8* promoter to non-hypoglycemic events in sulfonylureas-treated T2D patients	
			*KCNJ11* (/)	Promoter		
Cardona et al. (2019) [120]	^a^ Nested case-control study EWAS (methylation bead chip); ^b^ Replication testing EWAS (methylation bead chip)	^a^ Europeans from the EPIC-NORFOLK study (563/701) ^b^ Indian Asians from the LOLIPOP study (1074/1590) ^b^ Americans from the FHS study (403/2204)	*ABCG1* (cg06500161)	Body	RR (95%CI)	1.65 (1.45–1.89)
			*SREBF1* (cg11024682)	Body		1.56 (1.35–1.79)
			*TXNIP* (cg19693031)	3′-UTR		0.52 (0.46–0.6)
			*CPT1A* (cg00574958)	5′-UTR		0.69 (0.61–0.78)
Krause et al. (2019) [121]	Replication testing candidate (bisulfite pyrosequencing)	Europeans from the Northern Germans cohorts, Cohort 1 (176 control) Cohort 2 (100 obese)	*ABCG1* (cg06500161)	Body	Risk group stratification based on the combined methylation scores	
			*SREBF1* (cg11024682)	Body		
García-Calzón et al. (2020) [122] Part 1	^a^ Case-control study EWAS on the Discovery cohort (methylation bead chip) ^b^ Case-control study EWAS on the replication cohorts (methylation bead chip)	^a^ Europeans from the ANDIS study, responders/non-responders to metformin (26/21) ^b^ Europeans from the ANDIS study, responders/non-responders to metformin (48/39) ^b^ Europeans from the AN-DiU and OPTIMED cohorts, responders/non-responders to metformin (47/31)	/ (cg00153082)	Intergenic	Stratification of glycemic responders and non-responders to metformin therapy based on the combined methylation risk scores of the 11 CpG sites	
			*CFAP58* (cg03529510)	Body		
			*OR4S1* (cg05402062)	TSS1500		
			*GPHA2* (cg16704073)	Body		
			/ (cg01894192)	Intergenic		
			*SAP130* (cg16240962)	TSS1500		
			*SEPT11* (cg01070242)	5′-UTR/Body		
			/ (cg08713722)	Intergenic		
			*LRRN2* (cg05151280)	5′-UTR		
			*CST1* (cg07511259)	TSS1500		
			/ (cg01282725)	Intergenic		
García-Calzón et al. (2020) [122] Part 2	^a^ Case-control study EWAS on the Discovery cohort (methylation bead chip) ^b^ Case-control study EWAS on the replication cohorts (methylation bead chip)	^a^ Europeans from the ANDIS study, tolerant/intolerant to metformin (66/17) ^b^ Europeans from the ANDIS study, tolerant/intolerant to metformin (37/11) ^b^ Europeans from the AN-DiU and the OPTIMED cohorts, tolerant/intolerant to metformin (15/5)	*SCYL1* (cg27553780)	Body	Stratification of tolerant and intolerant individuals to metformin therapy based on the combined methylation risk scores of the 4 CpG sites	
			*FOXA2* (cg12356107)	TSS1500		
			*PGM1* (cg02994863)	1st Exon		
			*FAM107A* (cg08148545)	TSS200/Body		
Juvinao-Quintero et al. (2021) [124]	Meta EWAS EWAS (methylation bead chip)	Europeans from the ALSPAC, LBC1936, RSIII-1 and RS-Bios studies (340/3088)	*ABCG1* (cg06500161)	Body	RR (95% CI)	1.13 (1.06–1.21)
			*TXNIP* (cg19693031)	3′UTR		0.93 (0.89–0.98)
			*CPT1A* (cg00574958)	5′-UTR		0.79 (0.62–1.00)
			*HDAC4* (cg00144180)	5′-UTR		1.08 (1.01–1.16)
			*SYNM* (cg16765088)	Intergenic		0.93 (0.88–0.99)
			*miR23a* (cg24704287)	Intergenic		0.95 (0.89–1.02)

Abbreviations: ^a^, phase 1 of the study design; ^b^, phase 2 of the study design; RR, relative risk; 95% CI, 95% confidence interval; LOLIPOP study, London Life Sciences Prospective Population Study; KORA S3 and 4: Kooperative Gesundheitsforschung in der Region Augsburg Study 3 and Study 4; EPIC-Norfolk study, European Prospective Investigation into Cancer and Nutrition (EPIC)-Norfolk Study; FHS, Framingham Heart Study; ANDIS, All New Diabetics In Scania study; AN-DiU, All New Diabetics in Uppsala County; OPTIMED, Optimized program of personalized treatment of type 2 diabetes; ALSPAC, the Avon Longitudinal Study of Parents and Children; LBC1936, the Lothian Birth Cohort of 1936; RSIII-1 and RS-Bios, the Rotterdam Study III-1 and the Rotterdam Study-Biobank-Based Integrative Omics Studies. ^§^ Prospective cohort study: participants were non-diabetic at baseline and developed diabetes during follow-up (or not). Samples were investigated at the baseline.

## Data Availability

Not applicable.

## Appendix A

### Type 2 Diabetes: Overview

T2D is a complex metabolic disorder characterized by a dysregulation of nutrient metabolism [1]. It accounts for almost 90% of all known cases of diabetes mellitus [1]. Nearly half of all individuals with T2D are unaware of being affected [1]. The clinical hallmark for the diagnosis of T2D is hyperglycemia that is fasting glucose levels ≥ 126 mg·dL^−1^ or 2 h glucose levels ≥ 200 mg·dL^−1^ following a 75 g oral glucose challenge. Glycated hemoglobin A1C (HbA1c) levels ≥ 6.5% can also be used for the diagnosis of diabetes [161]. Pre-diabetes, which characterizes people with impaired fasting glucose (110–125 mg·dL^−1^) and/or impaired glucose tolerance (140–199 mg·dL^−1^) and/or HbA1c levels above 6.0% and below 6.4%, may precede T2D [163]. Indeed, disease progression from a pre-diabetic condition to T2D frequently occurs (conversion rate of 3–11% per year) [164].

Hyperglycemia, resulting from the defects in pancreatic β-cell function and peripheral resistance to insulin action in muscle, liver and adipose tissue, is important in determining the increased risk of developing many life-threatening complications in T2D patients such as microvascular (retinopathy, nephropathy and neuropathy) and macrovascular (accelerated atherosclerosis) comorbidities [2,13,15,165]. Activation of inflammatory pathways, pancreatic β-cell resistance to glucagon-like peptide 1, increased glucagon levels and enhanced hepatic sensitivity to glucagon, impaired insulin-mediated vasodilation, neurotransmitter dysfunction and increased renal glucose reabsorption also contribute to hampering the glycemic control in T2D patients [2,165].

Today, diabetes and its related complications can be effectively managed through [2]. Multiple antidiabetic medications, e.g., metformin, insulin sensitizers, drugs enhancing insulin secretion, GLP1 modulators, drugs targeting the intestinal or renal glucose absorption and insulin therapy, each one targeting pathophysiological defects of T2D, are available, and new avenues for treatment are currently in development [2]. Combining insulin therapy with other antidiabetic medications is helpful for glycemic control and favors insulin dose reduction [15,166,167]. Weight management often accompanies T2D therapy [168], and T2D can be prevented. Effective lifestyle interventions and good strategies to identify at-risk individuals have been demonstrated to represent powerful tools to have this objective accomplished [169]. How to use these tools to make the prevention of diabetes feasible remains to be established. In this area, the identification of reliable biomarkers holds credible promise.

## Appendix B

### Epigenome and Epigenetic Information

The phenotype in humans originates from combinations of qualitative and quantitative variations in gene transcription and is very much controlled by the independent and interacting effects of the genome and the environment [170]. Human diseases are consequences of variation in (i) the genotype (SNPs, rare gene variants, mutations or insertion/deletion sites or transposable element variations, which may impair the quality or the quantity of gene product or both); (ii) environmental events, which directly affect gene expression or contribute to post-translational modifications to the gene products; or (iii) results from a complex interplay among all these factors [170].

The Epigenome may affect phenotype as well [170]. The term “epigenome” describes the genome-wide distribution of a variety of molecular events, the “epigenetic information”, controlling the phenotype via regulation in gene expression [171,172,173]. In each individual, the epigenome may be distinct in different cell types, and in the same cells, they may change at different developmental stages [171,172,173]. Furthermore, the epigenome may differ during the progression of diseases, aging and following environmental cues [174].

Epigenetic information mainly includes mechanisms involved in the modification of chromatin such as DNA methylation, remodeling of chromatin structure and modification of histone tails (because of acetylation, methylation, ubiquitination and phosphorylation events), and mechanisms regulating gene expressions at the transcriptional, post-transcriptional, translational and post-translational levels, such as small RNAs and long non-coding RNAs [71]. In certain cases, epigenetic information may be inherited [71].
